# Gene methylation of human ovarian carcinoma stromal progenitor cells promotes tumorigenesis

**DOI:** 10.1186/s12967-015-0722-7

**Published:** 2015-11-23

**Authors:** Chih-Ming Ho, Daniel Tzu-bi Shih, Chih-Chiang Hsiao, Shih-Hung Huang, Shwu-Fen Chang, Wen-Fang Cheng

**Affiliations:** Department of Obstetrics and Gynecology, Gynecologic Cancer Center, Cathay General Hospital, Taipei, Taiwan; School of Medicine, Fu Jen Catholic University, Hsinchuang, New Taipei City, Taiwan; School of Medicine, Taipei Medical University, Taipei, Taiwan; Graduate Institute of Medical Sciences, School of Medicine, Taipei Medical University, #250 Wu-Hsing Street, Taipei, 110 Taiwan; Department of Pediatrics, Taipei Medical University Hospital, Taipei, Taiwan; Department of Pathology, Cathay General Hospital, Taipei, Taiwan; Department of Obstetrics and Gynecology, National Taiwan University, #7 Chung-Shan South Road, Taipei, 100 Taiwan; Graduate Institute of Oncology, National Taiwan University, Taipei, Taiwan; Graduate Institute of Clinical Medicine, College of Medicine, National Taiwan University, Taipei, Taiwan

**Keywords:** Tumorigenesis, Ovarian carcinoma stromal progenitor cells, 5-aza-2-deoxycytidine, Tumor suppressor genes, Methylation

## Abstract

**Background:**

This study aimed to investigate whether the DNA methylation of human ovarian carcinoma stromal progenitor cells (OCSPCs) could promote the tumorigenesis of ovarian carcinoma.

**Methods:**

OCSPCs were first isolated from fresh tumor tissues and ascites of ovarian cancer patients. In vivo and in vitro experiments on the effect of the OCSPCs on tumorigenesis and the effects of DNA demethylation on the OCSPCs were then performed.

**Results:**

The OCSPCs possessed self-renewal and multipotent differentiation capacity with elevated expressions of OCT4, NANOG, BMP2, BMP4, Rex-1, AC133 and TGF-β. The OCSPCs, when combined with tumor cells in vivo could promote tumor growth. The methylation profiles of tumor suppressor genes (TSGs) were significantly higher in the OCSPCs than in ovarian cancer cells (p < 0.001). 5-aza-2-dC could alter the methylation levels of TSGs in OCSPCs and also inhibit the tumor promoting capabilities of the OCSPCs by decreasing the proliferation of tumors cells. The expression levels of TSGs were re-expressed by 5-aza-2-dC to inhibit the self-renewal and growth of OCSPCs.

**Conclusions:**

OCSPCs with decreased TSG expressions in the ovarian tumor microenvironment were able to promote tumorigenesis which could be reversed by DNA demethylation. DNA demethylation reversing the expression of TSGs in OCSPCs may represent a potential therapeutic target for ovarian cancer.

**Electronic supplementary material:**

The online version of this article (doi:10.1186/s12967-015-0722-7) contains supplementary material, which is available to authorized users.

## Background

Ovarian cancer is the fifth leading cause of death from cancer in Western countries, and the leading cause of death from gynecologic cancer [1]. Most cases of ovarian cancer are high-grade serous carcinoma involving concurrent serous tubal intraepithelial carcinoma, which supports a clonal relationship characterized by *TP53* mutations [2–5]. Efforts at improving survival have focused on the early detection of ovarian cancer and on the development of new chemotherapeutic drugs. Therefore, it is essential to understand the initiation and mechanism underlying the progression of ovarian cancer.

At least one-third of epithelial ovarian cancers are associated with ascites, a massive amount of exudative fluid with a cellular fraction consisting mainly of cancer cells, lymphocytes, mesothelial cells, and soluble factors. Ascites is thought to contribute to the spreading of cancer cells to metastatic sites [6–8]. Stromal cells heterogeneously consist of fibroblasts, endothelial or mesothelial cells, adipocytes or adipose tissue-derived stromal cells, bone marrow-derived stem cells, and immunocytes. They can enhance tumor growth via secretion of growth or pro-angiogenetic factors such as fibroblast growth factor, vascular endothelial growth factor, and epidermal growth factor [9, 10]. Although ascites is a common symptom in patients with ovarian cancer, the origin of malignant ascitic fluid and its relationship to tumor progression are still poorly understood.

Recently, distinct DNA methylation profiles in ovarian serous neoplasms and their association with ovarian carcinogenesis and clinical outcome have been reported [11, 12]. The progression of ovarian cancer is associated with the accumulation of aberrant promoter methylation [12, 13], leading to transcriptional silencing of tumor suppressor genes (TSGs). Evidence suggests that genetic and non-genetic alterations in both ovarian surface epithelium and the surrounding stromal compartments may determine the phenotypic characteristics and functional performance of these cells. Preclinical and clinical studies have shown that hypomethylating agents can reverse platinum resistance in ovarian cancer cell lines and tumor xenografts [14–18]. In addition, aberrant TSG hypermethylation has been shown to be sufficient to transform somatic stem cells to fully malignant cells with cancer stem/initiating properties [19].

Mesenchymal stem cells (MSCs) can be recruited to the tumor microenvironment and are known as tumor-associated MSCs. Normal human bone marrow-derived MSCs can differentiate into tumor-associated fibroblasts which produce numerous growth factors to support angiogenesis, tumor growth and metastasis [20–22]. In addition, ovarian carcinoma-associated MSCs have been shown to promote tumor growth by increasing the number of cancer stem cells [23]. Thus, it is important to understand the phenotypic alteration of tumor-associated MSCs within the tumor and their contribution to tumorigenesis in patients with ovarian carcinoma. In this study, we isolated two types of ovarian cancer stromal progenitor cells (OCSPCs) (epithelial-like and mesenchymal-like cells) from ascites and cancerous tissues [24]. Cultured in vitro, these OCSPCs displayed the potential for self-renewal and long-term proliferation, and expressed the typical cancer stem/progenitor cell markers CD44high, CD24low, and AC133^+^ by in vitro culture. These OCSPCs also demonstrated high BMP-2, BMP4, TGF-b, Rex-1, and AC133 early gene expression, and expressed EGFR, integrin α2β1, CD146, and Flt-4, which are highly associated with tumorigenesis and metastasis. The epithelial-like OCSPCs demonstrated higher cytokeratin 18 and E-cadherin expression than the mesenchymal type cells. The mesenchymal type cells, in contrast, demonstrated higher AC133, CD73, CD105, CD117, EGFR, integrin a2b1, and CD146 surface marker expression than he epithelial type cells [24]. Genes methylation was significantly higher in the OCSPCs from ascites than that from tissues. OCSPCs can contribute to the progression of ovarian cancer accompanied by methylation of tumor suppressor genes.

## Methods

### Ascites and cancerous tissue sample collection from patients with epithelial ovarian cancer

The Institutional Review Board of our hospital approved the study protocol and all patients provided informed consent before the samples were collected. Tumor and ascites samples obtained during surgery were immediately taken to the laboratory for processing. Normal ovarian tissues were obtained from histologically-proven normal ovaries from patients with early-stage ovarian cancer.

### In vitro isolation and culture of OCSPCs from ascites and cancerous tissues

OCSPCs from ascites and cancerous tissues were isolated as described previously [24]. Briefly, the cells were pelleted by centrifugation at room temperature for 5 min at 1500 rpm for mesenchymal-like OCSPCs. For the selection of epithelial-like OCSPCs, Ficoll-Paque (GE Healthcare Life Sciences) gradient was applied to isolate mononuclear cells was and then washed with 2 mmol/L of EDTA. Total 3 × 10^6^ cells were re-suspended in culture medium [basal medium A: Dulbecco’s modified Eagle’s medium (DMEM/F12) supplemented with 10 % FBS (Hyclone), 10 ng/mL EGF and 10 ng/mL FGF-b1; basal medium B: M199 medium supplemented with 10 % FBS, 20 ng/mL EGF and 0.4 μg/mL hydrocortisone]. The cells were maintained in a humidified chamber with 5 % CO_2_ at 37 °C, and the media were refreshed every 3 days. OCSPCs at passage 4 were harvested for further experiments. Normal or cancerous ovarian tissue samples were minced in HBSS (Invitrogen; Grand Island, NY, USA), mixed with 1 mg/ml of collagenase 1A (Sigma) at 37 °C for 60 min, and then filtered through a 70-μm nylon mesh to remove the undigested tissue pieces and centrifuged to obtain cell pellets.

### Differentiating capabilities of ascites-derived OCSPCs

The protocols for adipogenic, osteogenic, chondrogenic, and neurogenic differentiation of the mesenchymal-like OCSPCs were as described previously [25]. The adipogenic differentiation medium was DMEM/LG with 10 % FBS, 0.5 mM isobutyl-methylxanthine, 1 μM dexamethasone, 10 μM insulin and 200 μM indomethacin. The osteogenic medium was DMEM/LG with 10 % FBS, 0.1 μM dexamethasone, 50 μM ascorbate-2-phosphate, and 10 mM β-glycerolphosphate. The chondrogenic medium was DMEM/LG with 1 % FBS, 6.25 μg/ml insulin, 10 ng/ml TGF-β1, and 50 nM ascorbate-2-phosphate. The neurogenic differentiation medium was DMEM/LG supplemented with 5 μg/ml insulin, 200 uM indomethacin, and 0.5 mM isobutyl-methylxanthine. The cells were fixed for histochemical staining after 14 days of adipogenic, osteogenic or chondrogenic differentiation, and the neurogenic-lineage cells were fixed for histochemical analysis after 28 days of differentiation. The angiogenic differentiation of the OCSPCs was analyzed by capillary formation using Matrigel. After being cultured for 7 days in EGM-2, the cells were trypsinized and plated onto a Matrigel coated (Matrigel:M199 = 1:1) 24-well plate. Capillary-like structures were observed by optical microscopy at the indicated time points.

### Flow cytometric analysis

Specific marker expressions in the OCSPCs were analyzed using flow cytometry (FACSCalibur, BD Biosciences). Fluorescein isothiocyanate- or phycoerythrin-conjugated antibodies against CA125, integrin α_2_β_1_, CD24, CD44, EGFR, CD105, CD34, CXCR4, SSEA1, SSEA3, SSEA4, Globo H, CD201, E-cadherin, AC133, CD73, CD90, CD117, CD146, CXCR4, PDGFR, NANOG, OCT3/4, cytokeratin 18, and FLT-4.

### Polymerase chain reaction (PCR) and sequence analysis of TP53

Exons 1-8 (except 9-11) of entire coding region of *TP*53 in both epithelial- and mesenchymal-like OCSPCs as well as ovarian cancer tissues were amplified and sequenced by PCR (Additional file 1: Table S1). The PCR products were subjected to electrophoresis on 2 % agarose gel, and BioEdit v7.1.3 software was used to analyze the DNA sequences. The obtained sequences were compared to published reference sequences (http://www.clinchem.org/content/44/7/1397.long).

### Immunocytochemistry of PAX8 and calretinin

Immunocytochemistry of both epithelial- and mesenchymal-like OCSPCs was performed using a Ventana Benchmark automated stainer (Ventana, Tucson, AZ, USA) with primary Abs against PAX8 and calretinin.

### Methylation-specific multiplex ligation-dependent probe amplification (MS-MLPA) of TSGs

MS-MLPA using SALSA MLPA kits including ME001B and ME003-A1 (MRC-Holland, Amsterdam, Netherlands) as described previously [26]. The methylation ratio (M-ratio) was calculated by dividing the relative peak value or probe fraction of the ligation-digestion sample by the relative peak value of the corresponding undigested ligation sample. M-ratio values of 0.00–0.29 were defined as being the absence of methylation. M-ratio values of 0.30–0.49 were defined as being mild methylation, 0.50–0.69 as moderate methylation, and >0.70 as extensive hypermethylation [27]. The cumulative methylation index (CMI) was calculated as the sum of the percentages of methylation for all genes [28].

### Sodium bisulfite treatment and methylation specific-PCR

Genomic DNA of the OCSPCs and tumors was isolated with a Genomic DNA kit (Geneaid Biotech, Bade City, Taiwan). The DNA was converted with sodium bisulfite using a CpGenome DNA modification kit (Millipore, MA, USA), purified and amplified by PCR with ThermoHotStart 2X Gold PCR Master mix (Applied Biosystems) with primers (Additional file 2: Table S2) at 95 °C for 10 min, followed by 40 cycles of 95 °C for 30 s, 62 °C for 30 s, and 72 °C for 40 s, with a final extension at 72 °C for 10 min and holding at 4 °C. Bisulfite-modified, *Sss* I-treated normal lymphocyte DNA served as the positive methylated control, and bisulfite-treated normal lymphocyte DNA served as the unmethylated control.

### RNA preparation and quantitative real time reverse transcriptase PCR (QRT-RT-PCR)

RNA was isolated by TriPure reagent (Roche), and stored at −80 °C prior to use. The quantity and quality of RNA were evaluated by spectrophotometric analysis. QRT-PCR was performed to quantitate the transcription of a specific gene. Total RNA was converted to cDNA with a Superscript III-reverse transcriptase kit (Invitrogen), and amplified by PCR with QIAGEN designed primers (Additional file 2: Table S2) at 95 °C for 5 min, followed by 40 cycles of 95 °C for 10 s, and 60 °C for 30 s. Roche Light Cycler Software 4.05 was used to analyze the data which were expressed as the mean of the expression level of the gene normalized by the GAPDH housekeeping gene.

QRT-PCR was performed to quantitate the effect of 5-aza-2-dC on gene expression. The OCSPCs were first treated with 10 μM of 5-aza-2-dC (Sigma, St. Louis, MO, USA). QRT-PCR was performed using an ABI Prism 7300 Sequence Detection System (Applied Biosystems) with Taqman Gene Expression Assay Hs00369360g1 and the primers as described above at 50 °C for 2 min, 95 °C for 10 min, and then 40 cycles of 95 °C for 15 s and 60 °C for 1 min. The interpolated number (C_t_) of cycles to reach a fixed threshold above the background noise was used to quantify amplification.

### Tumorsphere formation of OCSPCs

Adherent mesenchymal- or epithelial-like OCSPCs were cultured in DMEM/F12 containing 20 ng/mL bFGF, 20 ng/mL EGF, 10 ng/mL IGF, and 2 % B27 (Invitrogen, Carlsbad, CA, USA) with or without 5-aza-2-dC. The number of spheres was counted after 7 days under an Olympus light microscope. The tumorspheres obtained on 14 days were then harvested for FACS analysis.

### SKOV3 and SKOV3-Luc cell lines

The SKOV3 cells primarily obtained from ATCC were gifted by Professor CL Chang (Mackay-Memorial Hospital, Taipei, Taiwan), and SKOV3-Luc cells which were stably transduced with a luciferase-expressing lentivirus were used. The SKOV3 and SKOV3-luc cell lines were tested with human identification by STR marker to confirm the Cell line authentication. SKOV3 and SKOV3-luc cells were confirmed with ATCC Cell Bank DNA Profile (STR), and CSF1PO (11,11), TH01 (9,9.3), D13S317 (8,11), D16S539 (12,12), vWA (17,18), TPOX (8,11), Amelogenin (X,X), and D5S818 (11,11) markers with perfect match. Only the SKOV3-luc cells revealed a peak at D7S820 (13,14).

### In vivo animal experiments and tumor imaging

NOD/SCID mice were purchased from the National Animal Center (Taipei, Taiwan), and all experiments were approved by the Institutional Animal Care and Use Committee of Cathay General Hospital. The experiments (4 mice/group) were carried out with 1 × 10^6^ SKOV3-Luc cells alone or a 1:1 mixture of SKOV3-Luc cells with OCSPCs or normal ovarian stromal progenitor cells (NOSPCs) (either epithelial- or mesenchymal-like). Tumor growth was measured using calipers, and volumes were calculated based on the modified ellipsoid formula (*L* × *W* × *W*/2). Bioluminescence optical images (Xenogen IVIS 2000, Caliper Life Sciences) were obtained on day 12, 16, 22, 25, and 39 after tumor cell injection.

### Statistical analysis

Statistical analyses were performed using SPSS statistical software (SPSS 16.0.1 for Windows, Chicago, IL, USA). The frequencies of promoter methylation of 40 genes were compared using the Chi square test. The CMI and methylation of individual genes and the mRNA expression levels were assessed using the Mann–Whitney *U* test. A *p* value less than 0.05 was defined as being statistically significant.

## Results

### The expressions of differential markers in OCSPCs and NOSPCs

**Fig. 1:**
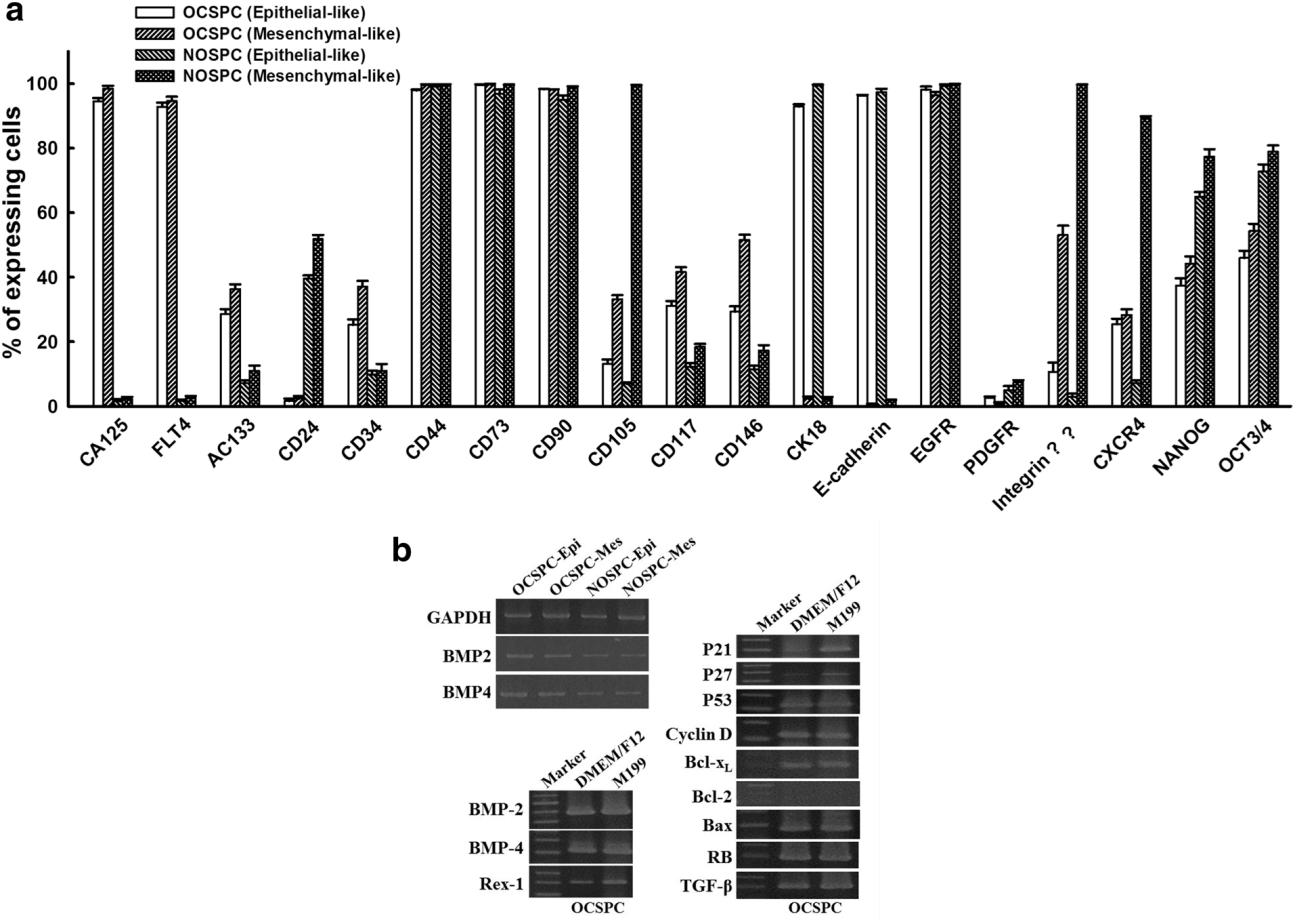
**a** The percentages of cells expressing typical cancer stem/progenitor cell markers and embryonic stem cell markers in the epithelial- and mesenchymal-like OCSPCs and NOSPCs analyzed by flow cytometry. **b** The expression levels of BMP-2, BMP4, TGF-β, Rex-1, RB, and AC133 cell cycle-related genes in P21, P27, and P53, cyclin D, Bcl-x_L_, and Bax in the epithelial and mesenchymal types of OCSPCs and NOSPCs by RT-PCR

The OCSPCs showed higher expressions of CA125, Flt4, AC133, CD34, CD117, and CD146, but lower expressions of CD24, NANOG and OCT3/4 compared to the NOSPCs (Fig. 1a). The expression levels of BMP2 and BMP4 were also higher in the OCSPCs (Fig. 1b). The OCSPCs also highly expressed cell cycle-related genes such as those encoding P21, P27, P53, cyclin D, and Bcl-x_L_ (Fig. 1b). The epithelial-like OCSPCs showed higher expressions of cytokeratin 18 and E-cadherin than the mesenchymal-like OCSPCs (Fig. 1a). In contrast, the mesenchymal-like OCSPCs had higher expressions of AC133, CD117, integrin α_2_β_1_, CD146, CXCR4, NANOG and OCT3/4 than the epithelial-like OCSPCs (Fig. 1a).

### Both epithelial- and mesenchymal-like OCSPCs originated from mesothelial cells not ovarian cancer cells

**Fig. 2:**
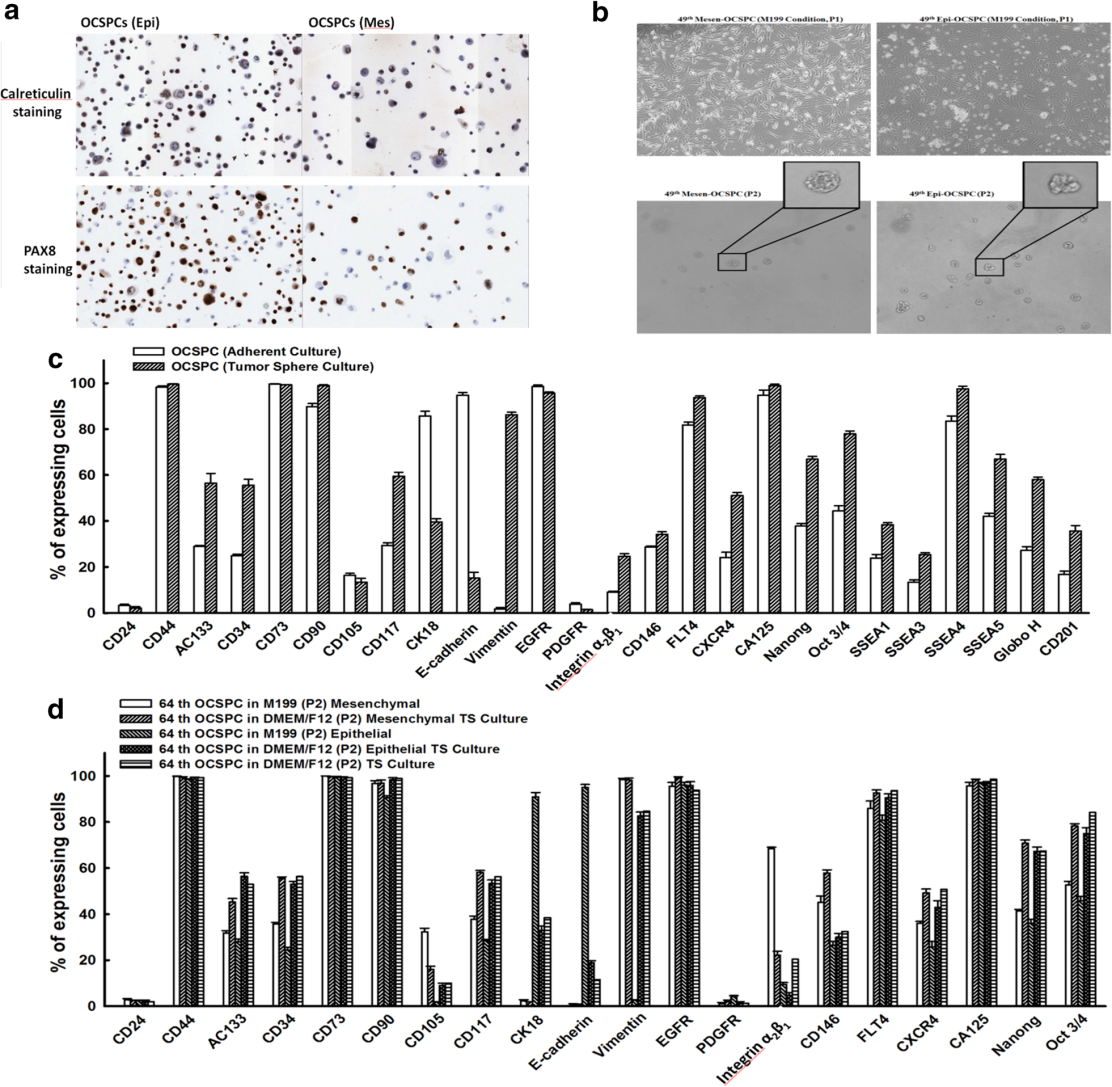
**a** Immunocytochemical analysis of PAX8 and Calretinin on both epithelial- and mesenchymal-like OCSPCs. **b** Cell morphology of original adherent epithelial- and mesenchymal-like OCSPCs and the respectively derived non-adherent floating tumorspheres. **c** The percentages of cells expressing various surface markers on non-adherent tumorspheres derived from either epithelial- or mesenchymal-like 49th OCSPCs analyzed by flow cytometry. **d** The percentages of cells expressing various surface markers on 64th OCSPCs cultured in either adherence conditions (selective for epithelial- and mesenchymal-like OCSPCs) or non-adherent (tumorsphere culture) and the respectively derived non-adherent tumorspheres analyzed by flow cytometry

Isolated epithelial- and mesenchymal-like OCSPCs in ascites and cancerous tissues were further analyzed to differentiate their origin. The G281T point mutation in exon 8 of *TP*53 (aspartic acid changed to tyrosine) was detected in the cancerous tissues (Additional file 3: Figure S1). However, only wild-type *TP*53 of both epithelial- and mesenchymal-like OCSPCs was noted. This indicated that the non-cancerous origin of the OCSPCs. Furthermore, immunocytochemical analysis demonstrated strong expressions of ovarian epithelial cancer cell and mesothelial cell markers, PAX8 and calretinin, in both types of OCSPC (Fig. 2a), implicating that a mesothelial origin of the OCSPCs was most likely.

### MS-MLPA profiles of TSG methylation in the OCSPCs and ovarian cancer cells

DNA methylation of TSGs in the OCSPCs and ovarian cancer cells was analyzed by MS-MLPA. The CMI among 40 TSGs was significantly higher in the OCSPCs from ascites than that from tissues (p < 0.001, Additional file 4: Table S3). The gene with the most frequent hypermethylation in the OCSPCs from ascites was CDKN2B (50 %), followed by RASSFIA (44 %) and DLC1 (44 %) (Additional file 5: Table S4). Whereas, the most frequently hypermethylated gene detected in the OCSPCs from cancerous tissues was CCND2 (50 %), followed by CDKN2B (25 %) and DLC1 (25 %) (Additional file 5: Table S4). Among these 40 genes, none was found to be methylated in the NOSPCs. The most frequently hypermethylated genes in the bulk tumors were CDKN2B (63 %), RASSFIA (50 %), and DLC1 (38 %) (Additional file 5: Table S4). In addition, the CMIs of CDKN2B, RASSFIA, DLC1 and CCND2 in the OCSPCs from ascites were significantly higher than those in the OCSPCs from tissues (p = 0.001) or bulk tumor cells (p = 0.038).

### Expression levels of CDKN2B, RASSFIA, DLC1 and CCND2 genes in the OCSPCs and ovarian cancerous tissues

To clarify whether the methylation status was correlated with the expression levels of CCND2, RASSF1A, DLC1 and CDKN2B, the mRNA levels of these genes were quantified by QRT-PCR. The mRNA levels of CCND2 (0.374 ± 0.433 vs. 0.733 ± 0.583, p = 0.012, student’s t test) and CDKN2B (0.143 ± 0.048 vs. 1.172 ± 0.740, p < 0.001, student’s t test), but not RASSF1A (1.635 ± 0.433 vs. 2.150 ± 1.630, p = 0.229, student’s t test) and DLC1 (1.269 ± 0.502 vs. 1.985 ± 1.099, p = 0.085, student’s t test), were significantly lower in the OCSPCs from ascites than those from bulk tumor tissues. When stratifying the OCSPCs by origin, the mRNA levels of CCND2 (0.074 ± 0.06 vs. 0.733 ± 0.583, p < 0.001, student’s t test), RASSF1A (0.553 ± 0.164 vs. 2.150 ± 1.630, p < 0.001, student t test) and CDKN2B (0.153 ± 0.056 vs. 1.172 ± 0.740, p < 0.001, student’s t test), but not DLC1 (1.471 ± 0.573 vs. 1.985 ± 1.099, p = 0.374, student t test) were significantly lower in the epithelial-like OCSPCs from ascites than those from bulk tumor tissues. Whereas, the mRNA levels of CDKN2B (0.128 ± 0.034 vs. 1.172 ± 0.740, p < 0.001, student’s t test) were significantly lower in the mesenchymal-like OCSPCs from ascites than those from bulk tumor tissues, but CCND2 (0.824 ± 0.325 vs. 0.733 ± 0.583, p = 0.441, student’s t test), RASSF1A (2.987 ± 0.872 vs. 2.150 ± 1.630, p = 0.066, student’s t test) and DLC1 (1.068 ± 0.357 vs. 1.985 ± 1.099, p = 0.051, student’s t test) remained similar in the mesenchymal-like OCSPCs from ascites than those from bulk tumor tissues. The mRNA levels of these four genes were significantly lower in the epithelial-like OCSPCs (0.604 ± 0.588 vs. 1.550 ± 1.280, p = 0.002, student’s t test) than those in the mesenchymal-like OCSPCs.

In addition, the mRNA levels of these four genes were significantly lower in the OCSPCs from ascites (1.038 ± 1.069 vs. 1.212 ± 0.902, p = 0.022, student’s t test) than those in the OCSPCs from tissues or those in the bulk tumor tissues (1.038 ± 1.069 vs. 1.509 ± 1.279, p < 0.001, student’s t test).

### Epithelial-like but not mesenchymal-like OCSPCs exhibited epithelial-mesenchymal transition (EMT) and formed tumorspheres

Both epithelial- and mesenchymal-like OCSPCs were able to form non-adherent, aggregated spheroids in low adherent discs supplemented with specific growth factors and could be maintained for at least 7 days (Fig. 2b). Only integrin α_2_β_1_ significantly increased in the cells within the tumorsphere compared to those in the original adherent OCSPCs (Figs. 1a, 2c). However, a higher expression of E-cadherin was detected in adherent epithelial-like OCSPCs than in the cells that formed non-adherent OCSPC spheroids (93.7 vs. 75 %) (Figs. 1a, 2c). However, similarly lower levels of E-cadherin were found in the mesenchymal-like non-adherent spheroid cells and the adherent OCSPCs (Figs. 1a, 2c). Furthermore, the glycan cell surface molecules, stage-specific embryonic antigen SSEA3 and SSEA4, were also highly expressed in the non-adherent spheroid cells (Fig. 2c). When the spheroids formed by both types of adherent OCSPCs were cultured for 14 days, the expression of E-cadherin decreased by at least fivefold (17.9 vs. 93.4 %), however vimentin increased by approximately 40-fold (83.1 vs. 2.4 %) in non-adherent spheroid cells compared to those in adherent epithelial-like OCSPCs (Fig. 2d), implicating the occurrence of EMT. E-cadherin remained at a similar lower level in the mesenchymal-like non-adherent spheroid cells and the adherent mesenchymal-like OCSPCs (Fig. 2d). The expressions of the other stemness markers including AC133, CD34, CD117, CXCR4, NANOG and OCT3/4 were also significantly increased in the non-adherent tumorspheres than those in the adherent OCSPCs (Fig. 2d).

### Multipotent capability of the OCSPCs to differentiate into various cell lineages

**Fig. 3:**
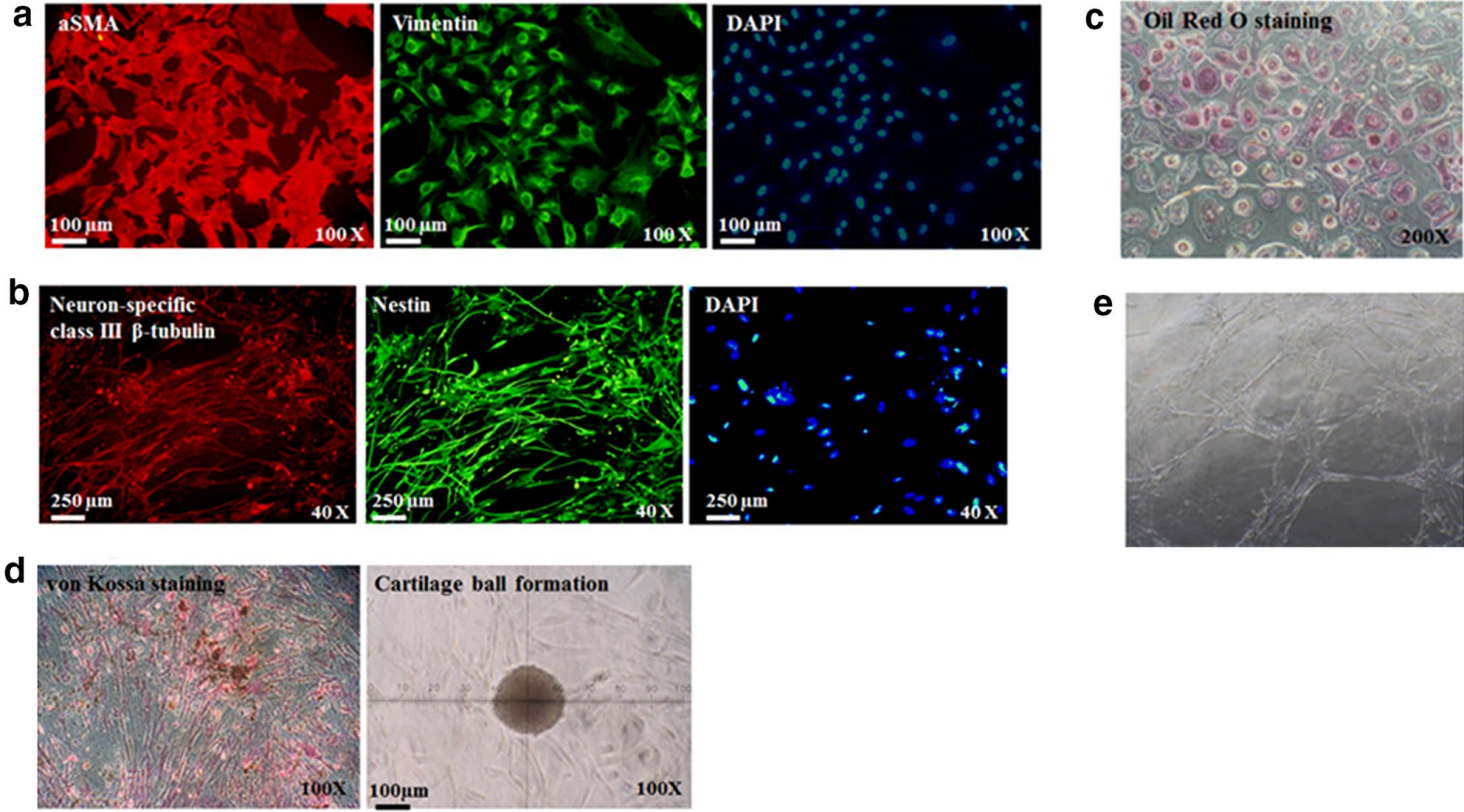
Multiple cell-lineage differentiation potential of OCSPCs. **a** Myofibroblast differentiation. The expressions of myofibroblast markers sSMA and vimentin. **b** Neurogenic differentiation. The neurogenic markers neuron-specific class II β-tubulin and nestin. **c** Adipogenic differentiation. Intracellular oil droplets were detected by Oil Red O staining. **d** Osteogenic and chondrogenic differentiation. Positive calcified extracellular matrix with von Kossa staining and cartilage ball formation. **e** Vascular differentiation. Vascular tube formation was noted on a Matrigel assay

The OCSPCs were further examined to see if they had the potential to produce different types of cells. The results showed that the mesenchymal-like OCSPCs had the capability to differentiate into multiple cell types including myogenic (Fig. 3a), neurogenic (Fig. 3b), adipogenic (Fig. 3c), osteogenic (Fig. 3d), chondrogenic (Fig. 3d), and vascular (Fig. 3e) cells under different conditional culture media.

### The OCSPCs promoted tumor growth in vivo

**Fig. 4:**
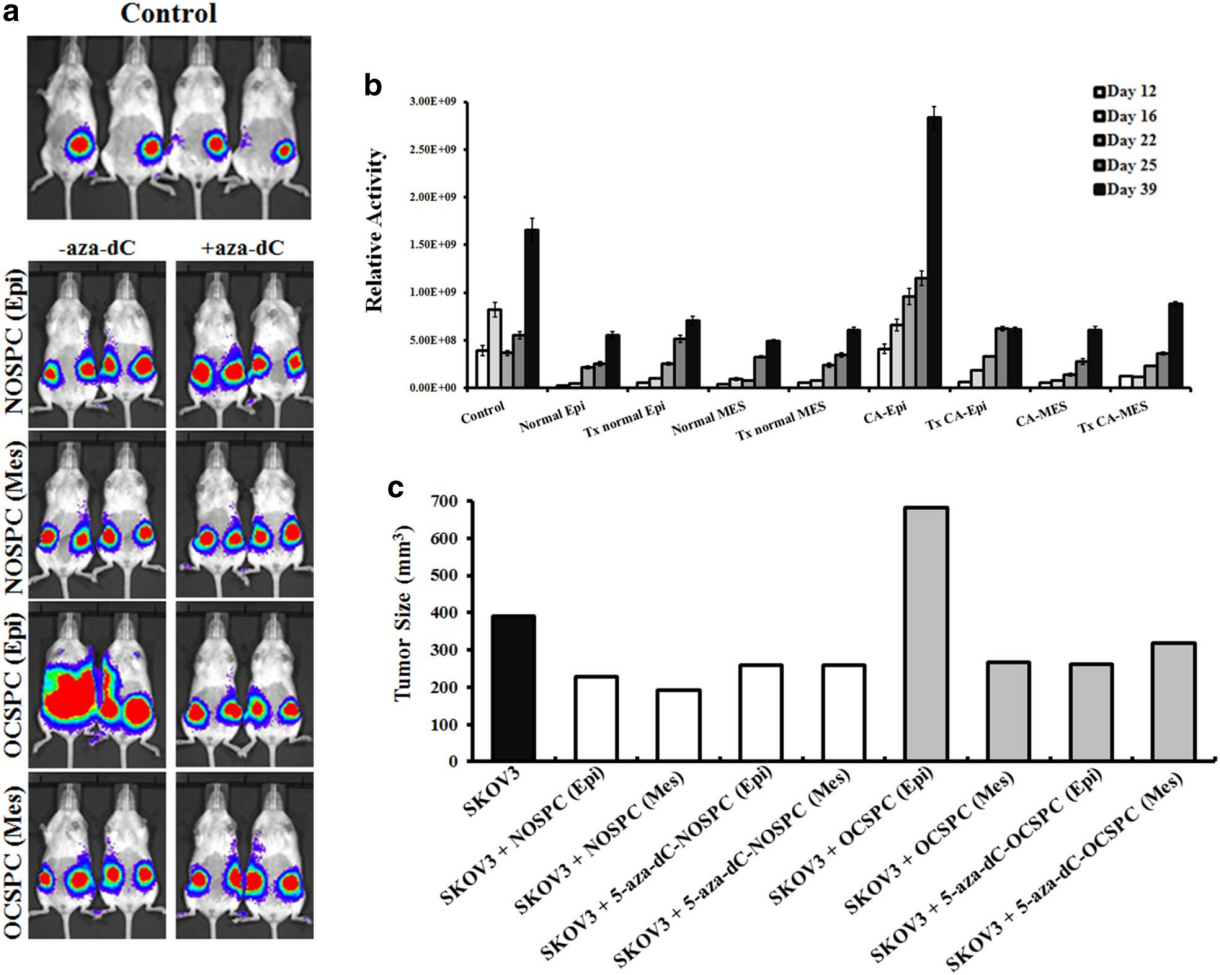
**a** Representative figures of chemiluminescent images of SKOV3-Luc tumor growth in various groups. **b** Relative luciferase activities of SKOV-3-Luc tumors in various groups at day 12, 16, 22, 25 and 39. Controls received SKOV3-Luc cells only. Normal represents NOSPCs, CA represents OCSPCs, Epi and Mes represent epithelial- or mesenchymal-like cells, respectively. **c** Sizes of the SKOV-3-Luc tumors in various groups at day 39

The influence of the OCSPCs on ovarian tumor growth was further evaluated with modified SKOV3 ovarian cancer cells, SKOV3-Luc cells carrying the luciferase reporter. The representative chemiluminescent images of SKOV3-Luc tumor growth in the various groups are shown in Fig. 4a. The mice that received SKOV3-Luc cells with epithelial-like OCSPCs demonstrated a consistent increase in luciferase activity compared with SKOV3-Luc cells alone, SKOV3-Luc cells with mesenchymal-like OCSPCs, and the SKOV3-Luc cells with NOSPCs (Fig. 4b). On day 39 after xenograft, the mean tumor sizes were largest in the mice that received SKOV3 cells with the epithelial-like OCSPCs compared with the other groups (Fig. 4c). These data suggest that epithelial-like OCSPCs can promote ovarian tumor growth. Beside, the histological analysis of these tumors was performed to reveal that all these tumors were similar to those in the histopathology of high grade serous adenocarcinoma of human ovarian cancer.

### 5-aza-2-dC changed the expression profiles and reactivated TSGs in the OCSPCs

**Fig. 5:**
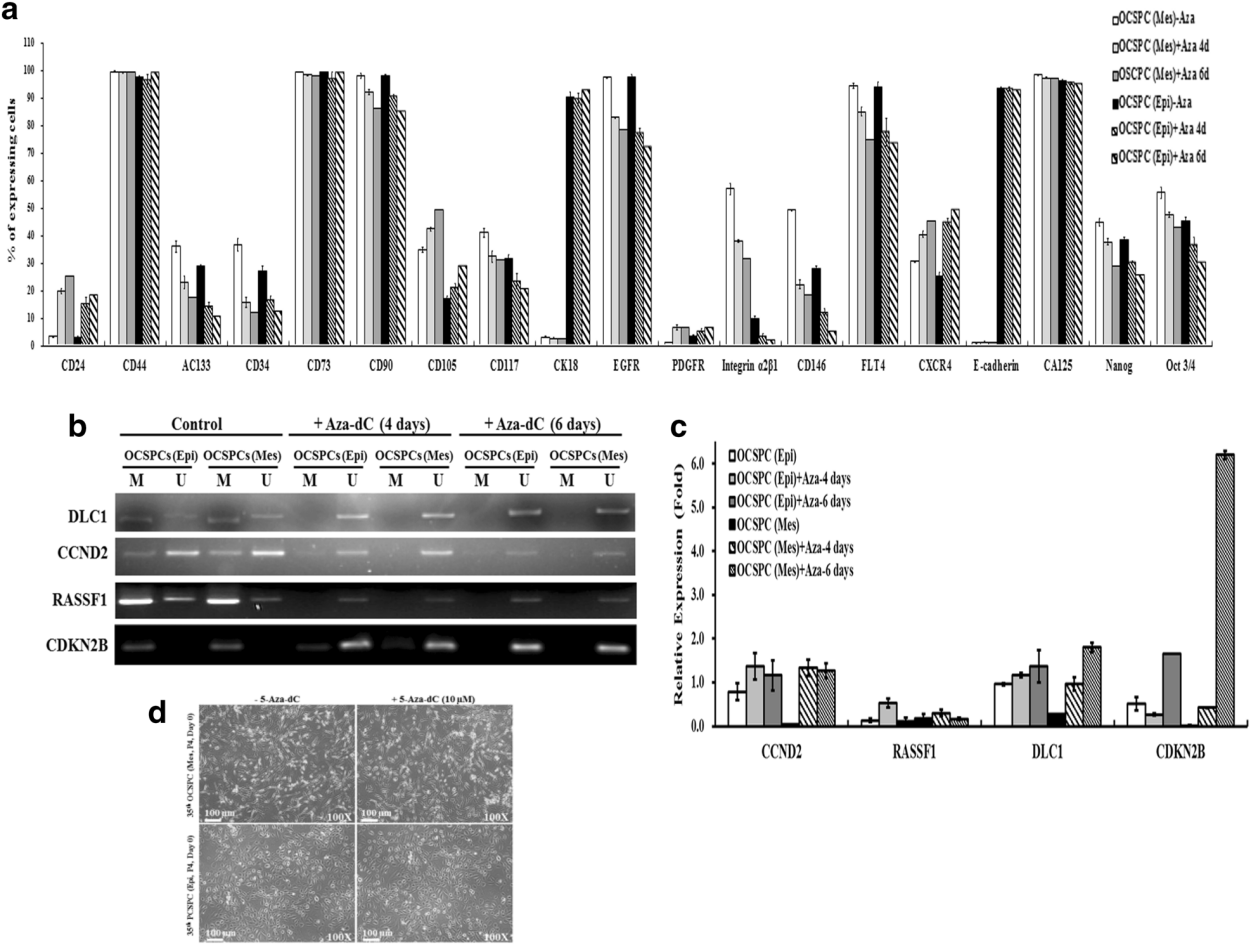
**a** The percentages of cells expressing various surface markers on OCSPCs treated with or without 5-aza-2-dC for 4 or 6 days as analyzed by flow cytometry. **b** Methylation status of DLC1, CCND2, RASSF1A, and CDKN2B genes treated with 5-aza-2-dC for 4 or 6 days by MS-PCR. **c** The expressions of CCND2, CDKN2B, RASSF1A, and DLC1 in OCSPCs treated with 5-aza-2-dC detected by quantitative real-time RT-PCR. **d** The morphology between mesenchymal or epithelial progenitor cells from malignant ascites treated with and without 5-aza-2-dC

To investigate the impact of demethylation on the tumorigenic promoting activity of OCSPCs, representative marker expressions were analyzed in cells treated with 5-aza-2-dC. The OCSPCs treated with 5-aza-2-dC had higher levels of CD24, CD105, and CXCR4, but lower levels of AC133, CD34, CD90, CD117, EGFR, integrin α_2_β_1_, CD146, FLT4, NANOG, and OCT3/4 compared to the cells without treatment (Fig. 5a). In addition, the CMI among the 40 TSGs tested was significantly lower in the epithelial-like OCSPCs after 5-aza-2-dC treatment compared to those without treatment (p < 0.001, Additional file 6: Table S5). However, the demethylating effect of 5-aza-2-dC was not detected in the mesenchymal-like OCSPCs, although demethylation (Fig. 5b) and increased RNA levels (Fig. 5c) of DLC1, CCND2, RASSF1A, and CDKN2B genes were identified in both the epithelial- and mesenchymal-like OCSPCs on day 4 or day 6 post-exposure to 5-aza-2-dC.

### 5-aza-2-dC altered self-renewal, growth- and stemness-related gene expressions in the OCSPCs and reduced the niche potential of OCSPCs for tumorigenicity of ovarian cancer

**Fig. 6:**
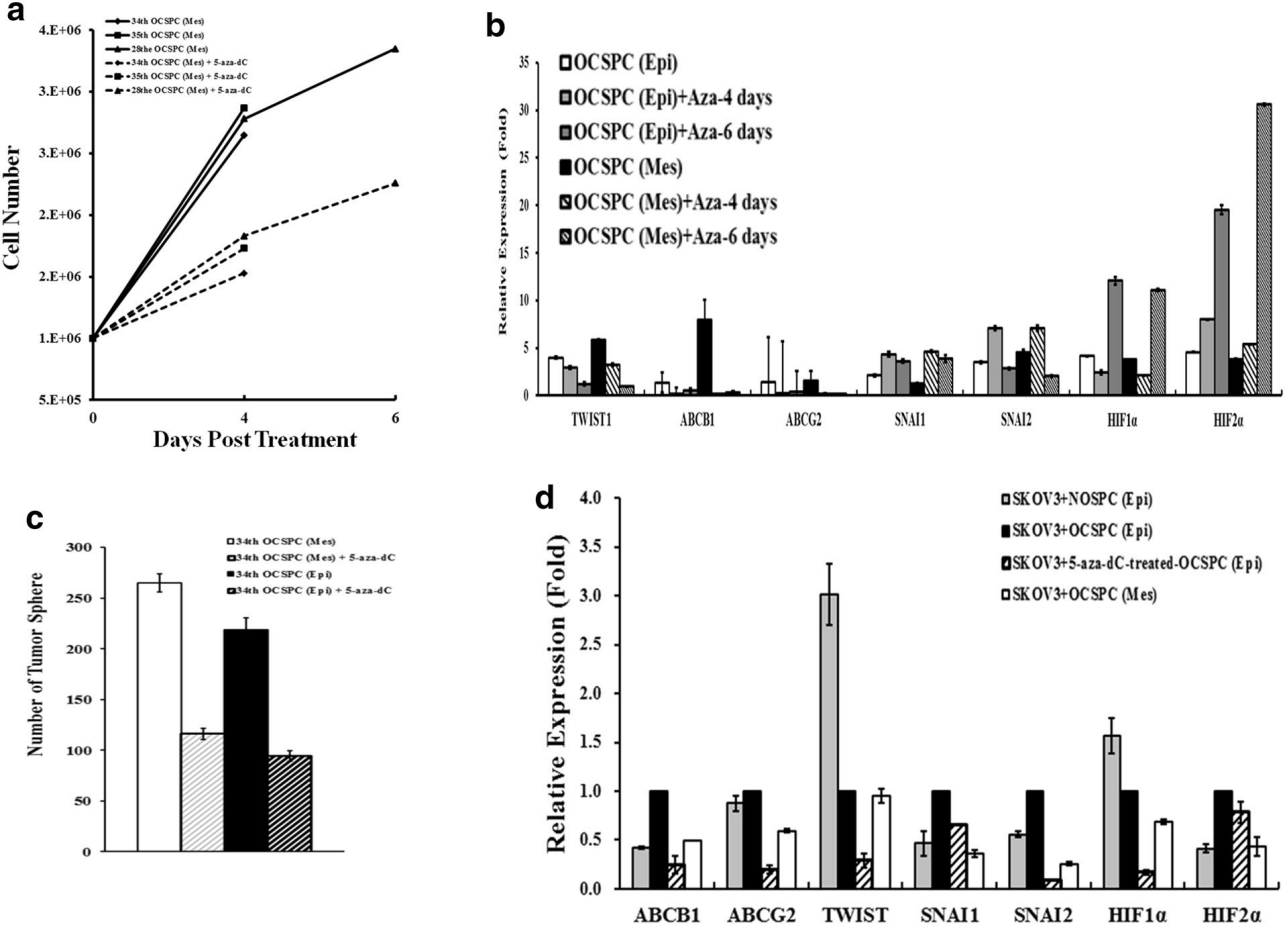
**a** Growth kinetics of epithelial- and mesenchymal-like OCSPCs derived from 28th, 34th and 35th OCSPCs treated with or without 5-aza-2-dC for 4 or 6 days. **b** The expressions of genes relating to EMT and drug resistance in OCSPCs from ascites of ovarian cancer patients treated with or without 5-aza-2-dC detected by quantitative real-time RT-PCR. **c** *Bar figure* of the number of tumor spheres in epithelial- and mesenchymal-like OCSPCs treated with or without 5-aza-2-dC. **d** Quantitative real-time RT-PCR analysis of the expressions of drug resistance, EMT and hypoxia-related genes in tumors isolated from xenograft mice of various groups

Neither mesenchymal- nor epithelial-like progenitor cells derived from malignant ascites had significant morphological changes upon 5-aza-2-dC treatment (Fig. 5d). Kinetic analysis demonstrated that the growth rates of the mesenchymal- or epithelial-like progenitor cells were reduced when treated with 5-aza-2-dC (Fig. 6a). The expressions of the stem-like cell surface markers including AC133, CD34, CD117, EGFR, integrin α_2_β_1_, Flt4, and CD146 in the OCSPCs were decreased upon 5-aza-2-dC treatment (Fig. 5a). In addition, both the epithelial- and mesenchymal-like OCSPCs showed significantly reduced levels of TWIST1, HIF-1α, MDR1, and ABCG2 on treatment with 5-aza-2-dC (Fig. 6b). The higher expression levels of MDR1 and ABCG2 efflux pumps may reflect increased drug resistance.

Exposure to 5-aza-2-dC also resulted in twofold decrease in the number of in vitro tumor spheres formed by the OCSPCs (Fig. 6c). The in vivo animal experiments revealed that the mice receiving SKOV3-Luc plus 5-aza-2-dC-treated epithelial-like OCSPCs demonstrated a consistent decrease in luciferase activity compared to the mice receiving SKOV3-Luc plus epithelial-like OCSPCs without 5-aza-2-dC for up to 39 days (Fig. 4a, b). However, 5-aza-2-dC treatment of the mesenchymal-like OCSPCs did not affect their influence on the growth of SKOV3-Luc tumor cells in the xenograft mice. In addition, 5-aza-2-dC treatment reduced the ability of the epithelial-like OCSPCs to promote tumorigenicity of SKOV3-Luc cancer cells and inhibited sphere formation of these OCSPCs. However, 5-aza-2-dC treatment also suppressed the tumorigenicity of SKOV3 in a mouse model [29].

### 5-aza-2-dC altered the expressions of genes related to EMT and drug resistance in the OCSPCs

To correlate the demethylating effect of 5-aza-2-dC with the expression levels of EMT- and drug resistance-related genes, tumors isolated from the xenograft mice were analyzed. The RNA levels of drug resistance-related genes MDR1 and ABCG2, and hypoxia-related genes HIF-1α and HIF-2α were reduced in tumors derived from SKOV3 with 5-aza-2-dC-treated epithelial-like OCSPCs compared to SKOV3 with epithelial-like OCSPCs without treatment, or with mesenchymal-like OCSPCs or normal progenitor cells (Fig. 6d). Sustained downregulation of key regulatory genes including TWIST, Slug and Snail, which promote the process of EMT, was decreased in the tumors generated by SKOV3 with 5-aza-2-dC-treated epithelial-like OCSPCs (Fig. 6d). These data support the hypothesis that epigenetic demethylating stem-like cells in the niche of ovarian cancer may reduce the tumorigenicity of epithelial ovarian cancer, possibly through the ablation of the EMT.

## Discussion

This is the first study to demonstrate that promoter methylation of TSGs not only in ovarian tumor tissues but also in cancer-associated stromal progenitor cells contributes to the progression of ovarian cancer. Given the present uncertainty regarding the precise phenotype of stromal progenitor cells, it is critical to understand the differences and similarities between stromal progenitor cells in primary tumors and in metastatic ascites. Our results showed that significantly decreased methylation levels correlated with higher expressions of certain TSGs (CCND2, RASSF1A, DLC1 and CDKN2B) in stromal progenitor cells from primary tumors compared to progenitor cells isolated from ascites. This finding supports our hypothesis that high methylation of the promoters of TSGs in progenitor cells which lose tumor suppressive function may contribute to tumor progression.

The biological characteristics of metastatic stem cells differ from those of stem cells in the primary tumor [30]. The current study showed similar methylation patterns in 40 TSGs between migrating stromal progenitor cells from ascites and those from primary tumor tissues, however the methylation levels in these two types of cells were different. It has been speculated that the malignant progression of stationary stromal progenitor cells from primary tumor tissues is through transformation into invasive and then migratory stromal progenitor cells from ascites which is influenced by significantly increasing methylation of specific TSGs. In this study, most of the 40 TSGs were defined as being polycomb-group genes, and the CMI among the 40 TSGs was significantly higher in stromal progenitor cells from ascites than those from tissues (p < 0.001). The most frequently hypermethylated genes in stromal progenitor cells from ascites and corresponding bulk tumor tissues from the patients with serous ovarian cancer at an advanced stage were CDKN2B, RASSFIA, DLC1, and CCND2. However, the CMIs of these four genes in stromal progenitor cells from ascites were significantly higher than those from tissues (p = 0.001) and bulk tumor cells (p < 0.05), and were inversely correlated with those of mRNA expression levels. Interestingly, the mRNA levels of the four genes were significantly lower in the epithelial-like stromal progenitor cells from ascites and tissues than those in the mesenchymal-like progenitor cells (p = 0.009).

The reversal of promoter DNA hypermethylation with restoration of the expressions of silenced genes is an attractive cancer therapy approach. Tsai et al. reported that transient low doses of DNA-demethylating agents exert durable antitumor effects on leukemia and breast cancer cells [31]. In addition, targeting the androgen receptor promoter in prostate stem/progenitor cells with 5-aza-2-dC has been reported to suppress prostate tumorigenesis [32]. It is well known that DNA methylation is one of the epigenetic mechanisms regulating the expression of stemness genes [33, 34] and the in vitro promotion of tumorsphere growth of cancer-associated MSCs [23]. To investigate whether 5-aza-2-dC could also affect the stemness of the identified stromal progenitor cells, QRT-PCR and flow cytometry analysis were used to characterize the expressions of OCT3/4 and NANOG in the OCSPCs. The results showed that the stromal progenitor cells had multipotent differentiation abilities (Fig. 3) with relatively abundant expressions (40–50 %) of OCT3/4 and NANOG which were significantly decreased after treatment with 5-aza-2-dC (Fig. 1a).

Our in vitro and in vivo experimental results demonstrated that 5-aza-2-dC inhibited the promoting capability of stromal progenitor cells for tumor growth by decreasing the proliferation of epithelial tumors cells and altering the methylation levels of TSGs in the stromal progenitor cells. Taken together, the demethylating agent, 5-aza-2-dC, reduced the growth rate and the expressions of stemness genes in human OCSPCs. To investigate whether a demethylating agent could also affect the behavior of these cancer stromal progenitor cells, 5-aza-2-dC was used to examine the expressions of EMT- and drug-resistance genes. Our results showed that these stromal progenitor cells had significantly higher levels of TWIST1, Slug (Snai2), Snail (Snai1), MDR1 (ABCB1), and ABCG2 genes, which are involved in the EMT and drug resistance, than those in normal controls (data not shown).

The antitumor responses we demonstrated may involve a resetting of the abnormal epigenetic status in stromal progenitor cells for their interaction with cancer cells. It is important to note that altered gene expression patterns were accompanied by antitumor effects, and 5-aza-2-dC treatment did not kill the cells. Thus, 5-aza-2-dC may cause sustained alterations in multiple key pathways involved in tumorgenesis, such as downregulation of the EMT, stemness, and drug resistance-related genes, and this effect may continue even after treatment is stopped. This finding may help in the development of a therapeutic approach for ovarian cancer treatment. Indeed, targeting stem/progenitor cells has emerged as a novel potential approach to battle ovarian cancer [34]. The advantages of using 5-aza-2-dC to target stromal progenitor cells include effectively suppressing their self-renewal/proliferation ability and decreasing the proliferation of tumors cells. In addition, few side effects have been reported compared to the toxicity induced by other approaches to target stem/progenitor cells [35, 36], and the durable inhibition of tumor growth (lasting for at least 6 weeks in this study). Importantly, this approach can be used as combination therapy with conventional cytotoxic drugs or as maintenance therapy, suppressing most of the tumor cells containing stromal progenitor cells yet overcoming drug resistance of stem/progenitor cells and rescuing TSG function.

Accumulating evidence indicates that prostate cancer and metastasis-initiating cells expressing stem-like markers including CD133^+^, CD44^high^, ALD^high^, ABCG2^+^ and CXCR4^+high^ and endowed with a high self-renewal ability are critical for prostate cancer progression, metastasis and resistance to current clinical therapies [32, 37]. In this study, the OCSPCs expressed similar stem cell-like markers such as CD133^+^, CD44^high^ and CXCR4^+^, and more specifically those expressing CD73^high^, CD117^+^, Flt4^high^, OCT3/4^+^, and NANOG^+^ possessed similar activity. Although numerous reports have focused on cancer stem cells in primary tumors [38], studies on cancer stem cells or MSCs and stromal progenitor cells in metastasis have just begun to emerge. To date, very few studies have focused on human solid tumor-associated MSCs. Our previous study demonstrated that stromal progenitor cells (tumor-associated stromal/progenitor cells) are present not only in human ovarian cancer tissues but also in ascites [24], which is consistent with other reports that cancer-associated mesenchymal stem cells (CA-MSCs) are universally present in human ovarian cancer [23].

Our results support that mesenchymal-like cells are non-tumorigenic. However, epithelial-like cells may be tumorigenic with cancer stem-like characteristics which may enhance SKOV3-Luc tumor growth and recapitulate in the original tumor. Both cell populations in this study were selected and cultured as adherent cells and formed non-adherent aggregated tumor spheroids under suitable conditions. Importantly, the expression of E-cadherin was significantly down-regulated and vimentin expression was significantly upregulated when adherent epithelial-like OCSPCs grew as non-adherent spheroids (94.5 vs. 18.7 % and 2.1 vs. 83.1 %, respectively), while the levels of these two genes remained unchanged in the mesenchymal-like non-adherent spheroids and the adherent mesenchymal-like OCSPCs. Moreover, non-adherent spheroid cells derived from adherent epithelial-like OCSPCs displayed a mesenchymal phenotype, similar to non-adherent spheroid cells from ascites. The epithelial-like OCSPCs were shown to be capable of promoting tumor growth with an increasing tumor size, while the mesenchymal-like OCSPCs did not. These results suggest that epithelial-like progenitor cells may exhibit partial EMT and form tumorspheres which are more characteristic of cancer stem-like cells under low adherent and serum free conditions. Besides, adherent epithelial-like stromal progenitor cells may originate and transform from non-adherent tumorspheres via the mesenchymal-epithelial transition (MET) under serum induction with some loss of stemness. However, the mesenchymal-like progenitor cells did not follow the MET. The expressions of several mesenchymal cell surface markers (CD73, CD90, CD29, CD146, CD105 and integrin α_2_β_1_) and their ability to interact with tumor cells suggest that epithelial-like progenitor cells exhibit similar features to those of MSCs.

Our results support that adherent epithelial-like stromal progenitor cells may originate and transform from non-adherent tumorspheres via the mesenchymal-epithelial transition (MET) under serum induction with some loss of stemness. However, it is better to validate our results by ex vivo experiment such as isolating non-adherent tumorspheres from ascites of ovarian cancer patient, and then culturing them under serum induction.

A phase II trial concluded that epigenetic modulation of DNA methylation restores platinum sensitivity in patients with platinum-resistant ovarian cancer, resulting in significant clinical activity as evidenced by a high response rate and prolonged progression-free survival [27, 39]. These findings support our hypothesis that demethylating agents not only restore sensitivity to platinum-based treatment in patients with platinum-resistant ovarian cancer, but also restore the function of TSGs in stromal progenitor cells to help inhibit tumor growth. The expressions of MDR1 and ABCG2 were significantly reduced in the stromal progenitor cells after 5-aza-2-dC treatment, implying that this treatment may overcome drug resistance of stem/progenitor cells. The number of tumorspheres was also significantly reduced in stromal progenitor cells after 5-aza-2-dC treatment, further suggesting that this treatment may decrease cell stemness.

## Conclusions

We have shown that OCSPCs possessed self-renewal and multipotent differentiation capacity by elevating the expressions of OCT4 and NANOG, BMP2, BMP4, Rex-1, AC133 and TGF-β and with high methylation profiles of TSGs. Besides, OCSPCs, when combined with tumor cells in vivo, could promote tumor growth. The demethylating agent, 5-aza-dC, could alter the methylation levels of TSGs in OCSPCs and then inhibited the tumor promoting capabilities of OCSPCs. OCSPCs can be potential targets for the treatment of ovarian carcinoma. The most value of the study is the new finding that the methylation levels of metastatic stem cells in ascites is different from those in primary tumor. To discover the biological characteristics of metastatic stem cells needs more explorations through experiments.

## Additional files

10.1186/s12967-015-0722-7 Primer sequences in Exon 1-8 of *TP53* gene.
10.1186/s12967-015-0722-7 Primer sequences used for MS-PCR.
10.1186/s12967-015-0722-7 Sequencing analysis of the G281 point mutation in exon 8 of *P*53 in the cancerous tissues, and epithelial- and mesenchymal-like OCSPCs. G (aspartic acid) to T (tyrosine) point mutation was noted in the cancerous tissues, but not in both epithelial- and mesenchymal-like OCSPCs.
10.1186/s12967-015-0722-7 The CMI among 40 TSGs in ascites and ovarian cancerous tissues.
10.1186/s12967-015-0722-7 Frequency of methylation among stromal progenitor cells from ascites, cancerous tissues, and bulk tumor cells.
10.1186/s12967-015-0722-7 The CMI among the 40 TSGs in the epithelial-like OCSPCs treated with or without 5-aza-2-dC.
