# Supplementary material for: Gene methylation of human ovarian carcinoma stromal progenitor cells promotes tumorigenesis
Source: J Transl Med. 2015 Nov 23;13:367. doi: 10.1186/s12967-015-0722-7 (PMC4655458; doi:10.1186/s12967-015-0722-7)
Supplement: Supplementary file 1 — 10.1186/s12967-015-0722-7 Primer sequences in Exon 1-8 of TP53 gene. [file 12967_2015_722_MOESM1_ESM.docx]

Table S1. Primer sequences in Exon 1-8 of *TP53* gene

| Code No | Gene Name | Comment | Reference | Sequence(5’to 3’) | Length (nts) | TM |
| --- | --- | --- | --- | --- | --- | --- |
| BC-461  BC-462 | Homo sapiens tumor  protein p53(TP53) exon05 | sense  antisense | NT_010718  NT_010718 | TCTGTCTCCTTCCTCTTCCTACA  AACCAGCCCTGTCGTCTCT | 23  19 | 59.51  59.85 |
| BC-463  BC-464 | Homo sapiens tumor  protein p53(TP53) exon06 | sense  antisense | NT_010718  NT_010718 | CAGGCCTCTGATTCCTCACT  CTTAACCCCTCCTCCCAGAG | 20  20 | 59.4  60.06 |
| BC-465  BC-466 | Homo sapiens tumor  protein p53(TP53) exon07 | sense  antisense | NT_010718  NT_010718 | CTTGGGCCTGTGTTATCTCC  GGGTCAGAGGCAAGCAGA | 20  18  21  20 | 59.55  60.07  59.31  60.11 |
| BC-467  BC-468 | Homo sapiens tumor  protein p53(TP53) exon08 | sense  antisense | NT_010718  NT_010718 | GGGACAGGTAGGACCTGATTT  TAACTGCACCCTTGGTCTCC |  |  |
| exon p53-1F  exon p53-1R | Homo sapiens tumor  protein p53(TP53) exon01 | sense  antisense | NT_010718  NT_010718 | ACTTGTCATGGCGACTGTCC  CTCAGAGAGGACTCATCAAGT | 20  21 | 53.8  52.4 |
| exon p53-234F  exon p53-234R  exon p53-234FF2 | Homo sapiens tumor  protein p53(TP53) exon2-4 | sense  antisense  sense | NT_010718  NT_010718  NT_010718 | TGTCTCATGCTGGATCCCCA  GTCTCATGGAAGCCAGCCC  CAGCCATTCTTTTCCTGCTC | 20  19  20 | 53.8  55.4  51.8 |
