# Supplementary material for: Gene methylation of human ovarian carcinoma stromal progenitor cells promotes tumorigenesis
Source: J Transl Med. 2015 Nov 23;13:367. doi: 10.1186/s12967-015-0722-7 (PMC4655458; doi:10.1186/s12967-015-0722-7)
Supplement: Supplementary file 2 — 10.1186/s12967-015-0722-7 Primer sequences used for MS-PCR. [file 12967_2015_722_MOESM2_ESM.docx]

Table S2. Primer sequences used for MS-PCR

| Gene | Comment | Sequence(5’to 3’) |
| --- | --- | --- |
| methylated CCND2 | Forward  Reversed | GGAGGTGAAGAAACGTTATTAGATC  GAAAACATAAAACCTCCACGCT |
| unmethylated CCND2 | Forward  Reversed | AGGTGAAGAAATGTTATTAGATTGT  CAAAAACATAAAACCTCCACACT |
| methylated RASSF1A | Forward  Reversed | GAGAGCGCGTTTAGTTTCGTTTTC  CCGTACTTCGCTAACTTTAAACGCT |
| unmethylated RASSF1A | Forward  Reversed | AGAGTGTGTTTAGTTTTGTTTTTGG  ATTAAACCCATACTTCACTAACTTTAAACA |
| methylated CDKN2B | Forward  Reversed | TATGTTTAGTGGGGGCGGTAGC  GAACAACATCATACACCGATCGAA |
| unmethylated CDKN2B | Forward  Reversed | GTATGTTTAGTGGGGGTGGTAGTGA  CAAACAACATCATACACCAATCAAA |
| methylated DLC1 | Forward  Reversed | TTTTGTGATTTTTGTTTTTGTATTC  TTAACGACGAACTATTCTCCGAC |
| unmethylated DLC1 | Forward  Reversed | TTTGTGATTTTTGTTTTTGTATTTG  CCTTAACAACAAACTATTCTCCAAC |
