# Supplementary figures and images for: Gene methylation of human ovarian carcinoma stromal progenitor cells promotes tumorigenesis
Source: J Transl Med. 2015 Nov 23;13:367. doi: 10.1186/s12967-015-0722-7 (PMC4655458; doi:10.1186/s12967-015-0722-7)

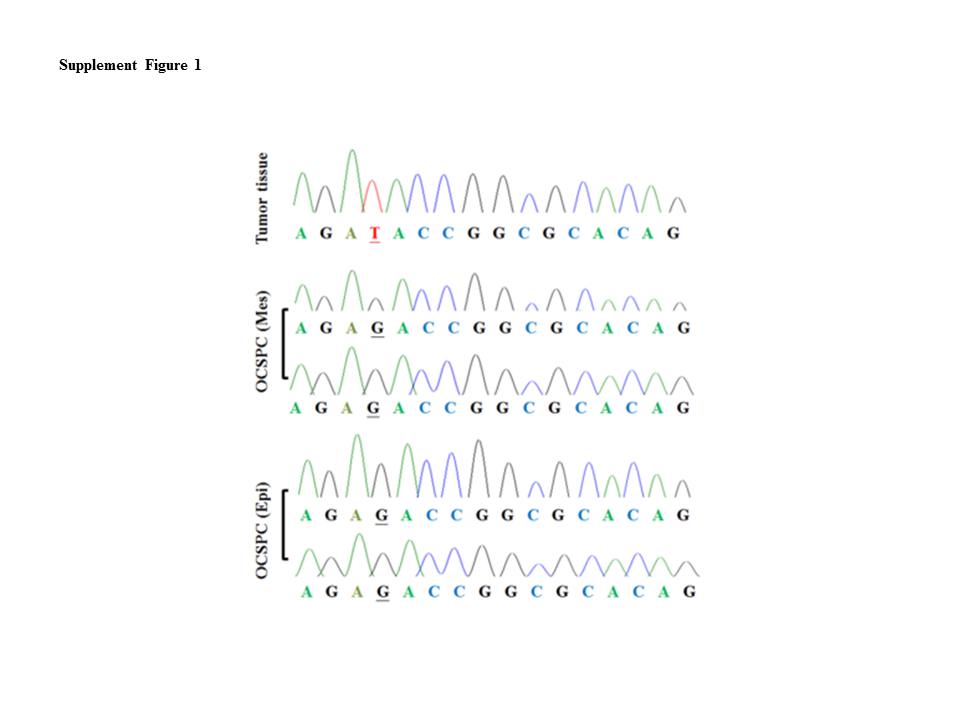

Supplement: Supplementary file 3 — 10.1186/s12967-015-0722-7 Sequencing analysis of the G281 point mutation in exon 8 of P53 in the cancerous tissues, and epithelial- and mesenchymal-like OCSPCs. G (aspartic acid) to T (tyrosine) point mutation was noted in the cancerous tissues, but not in both epithelial- and mesenchymal-like OCSPCs. [file 12967_2015_722_MOESM3_ESM.tiff]
