# Supplementary material for: Gene methylation of human ovarian carcinoma stromal progenitor cells promotes tumorigenesis
Source: J Transl Med. 2015 Nov 23;13:367. doi: 10.1186/s12967-015-0722-7 (PMC4655458; doi:10.1186/s12967-015-0722-7)
Supplement: Supplementary file 4 — 10.1186/s12967-015-0722-7 The CMI among 40 TSGs in ascites and ovarian cancerous tissues. [file 12967_2015_722_MOESM4_ESM.doc]

Table S3. The CMI among 40 TSGs in ascites and ovarian cancerous tissues

| **ME003-A1 MLPA** | **NO.1** | | | **NO.2** | | | **NO.3** | | |
| --- | --- | --- | --- | --- | --- | --- | --- | --- | --- |
| **ME001-C1 MLPA** | **T** | **AE** | **AM** | **T** | **AE** | **AM** | **T** | **AE** | **AM** |
| PRDM2 | 0.01 | 0.01 | 0.02 | 0 | 0.10 | 0.04 | 0.05 | 0.03 | 0.01 |
| RUNX3 | 0.05 | 0.06 | 0.10 | 0.17 | 0.17 | 0.14 | 0.08 | 0.14 | 0.11 |
| RARB | 0.08 | 0.07 | 0.07 | 0.13 | 0.10 | 0.17 | 0.08 | 0.10 | 0.12 |
| HLTF | 0.01 | 0.00 | 0.00 | 0.06 | 0.02 | 0.01 | 0.01 | 0.01 | 0.01 |
| SCGB3A1 | 0.04 | 0.10 | 0.07 | 0.17 | 0.20 | 0.10 | 0.11 | 0.10 | 0.06 |
| ID4 | 0.03 | 0.04 | 0.03 | 0.06 | 0.08 | 0.11 | 0.09 | 0.10 | 0.10 |
| TWIST1 | 0.01 | 0.00 | 0.00 | 0.08 | 0.01 | 0.01 | 0.01 | 0.02 | 0.01 |
| SFRP4 | 0.03 | 0.13 | 0.02 | 0.03 | 0.07 | 0.05 | 0.04 | 0.04 | 0.04 |
| DLC1 | 0.19 | 0.09 | 0.28 | 0.33 | 0.45 | 0.41 | 0.21 | 0.48 | 0.38 |
| SFRP5 | 0.06 | 0.01 | 0.07 | 0.06 | 0.09 | 0.06 | 0.13 | 0.05 | 0.03 |
| BNIP3 | 0.08 | 0.08 | 0.05 | 0.06 | 0.06 | 0.03 | 0.06 | 0.06 | 0.03 |
| H2AFX | 0.02 | 0.01 | 0.04 | 0.09 | 0.12 | 0.07 | 0.06 | 0.05 | 0.02 |
| CCND2 | 0.11 | 0.19 | 0.19 | 0.38 | 0.33 | 0.36 | 0.28 | 0.27 | 0.17 |
| CACNA1G | 0.01 | 0.01 | 0.02 | 0.15 | 0.08 | 0.03 | 0.04 | 0.04 | 0.01 |
| TGIF | 0.00 | 0.00 | 0.00 | 0.00 | 0.01 | 0.00 | 0.00 | 0.00 | 0.00 |
| BCL2 | 0.00 | 0.01 | 0.08 | 0.20 | 0.01 | 0.00 | 0.00 | 0.00 | 0.01 |
| CACNA1A | 0.10 | 0.01 | 0.00 | 0.10 | 0.03 | 0.01 | 0.27 | 0.01 | 0.01 |
| TP73 | 0.03 | 0.12 | 0.05 | 0.07 | 0.05 | 0.04 | 0.10 | 0.05 | 0.09 |
| CASP8 | 0.10 | 0.08 | 0.01 | 0.02 | 0.10 | 0.07 | 0.05 | 0.08 | 0.06 |
| VHL | 0.00 | 0.02 | 0.00 | 0.07 | 0.00 | 0.00 | 0.01 | 0.00 | 0.01 |
| RARB | 0.02 | 0.57 | 0.01 | 0.13 | 0.00 | 0.03 | 0.05 | 0.00 | 0.04 |
| MLH1 | 0.07 | 0.14 | 0.04 | 0.03 | 0.01 | 0.09 | 0.09 | 0.04 | 0.07 |
| RASSF1 | 0.30 | 0.13 | 0.02 | 0.31 | 0.47 | 0.54 | 0.08 | 0.64 | 0.54 |
| FHIT | 0.04 | 0.02 | 0.01 | 0.00 | 0.03 | 0.06 | 0.05 | 0.04 | 0.04 |
| APC | 0.04 | 0.00 | 0.01 | 0.03 | 0.03 | 0.05 | 0.09 | 0.03 | 0.05 |
| ESR1 | 0.02 | 0.07 | 0.07 | 0.03 | 0.11 | 0.20 | 0.07 | 0.13 | 0.14 |
| CDKN2A | 0.30 | 0.18 | 0.33 | 0.16 | 0.53 | 0.69 | 0.44 | 0.45 | 0.43 |
| DAPK1 | 0.01 | 0.11 | 0.06 | 0.00 | 0.07 | 0.14 | 0.05 | 0.11 | 0.26 |
| PTEN | 0.04 | 0.20 | 0.07 | 0.09 | 0.08 | 0.06 | 0.08 | 0.05 | 0.08 |
| CD44 | 0.02 | 0.03 | 0.03 | 0.00 | 0.04 | 0.06 | 0.09 | 0.05 | 0.08 |
| GSTP1 | 0.19 | 0.01 | 0.08 | 0.03 | 0.07 | 0.06 | 0.06 | 0.08 | 0.07 |
| ATM | 0.15 | 0.09 | 0.14 | 0.04 | 0.13 | 0.12 | 0.15 | 0.11 | 0.16 |
| IGSF4 | 0.01 | 0.01 | 0.00 | 0.02 | 0.04 | 0.08 | 0.07 | 0.04 | 0.07 |
| CDKN1B | 0.02 | 0.12 | 0.03 | 0.08 | 0.03 | 0.04 | 0.06 | 0.04 | 0.06 |
| CHFR | 0.02 | 0.09 | 0.01 | 0.04 | 0.14 | 0.08 | 0.04 | 0.08 | 0.04 |
| BRCA2 | 0.00 | 0.11 | 0.00 | 0.02 | 0.00 | 0.02 | 0.04 | 0.01 | 0.02 |
| CDH13 | 0.13 | 0.10 | 0.09 | 0.14 | 0.09 | 0.11 | 0.14 | 0.13 | 0.15 |
| HIC1 | 0.00 | 0.24 | 0.00 | 0.16 | 0.01 | 0.04 | 0.05 | 0.01 | 0.05 |
| BRCA1 | 0.00 | 0.16 | 0.00 | 0.07 | 0.01 | 0.03 | 0.05 | 0.01 | 0.08 |
| TIMP3 | 0.19 | 0.33 | 0.12 | 0.22 | 0.17 | 0.26 | 0.17 | 0.20 | 0.13 |
| **CMI** | **2.53** | **3.75** | **2.21** | **3.81** | **4.15** | **4.45** | **3.58** | **3.89** | **3.83** |
