# Supplementary material for: Gene methylation of human ovarian carcinoma stromal progenitor cells promotes tumorigenesis
Source: J Transl Med. 2015 Nov 23;13:367. doi: 10.1186/s12967-015-0722-7 (PMC4655458; doi:10.1186/s12967-015-0722-7)
Supplement: Supplementary file 5 — 10.1186/s12967-015-0722-7 Frequency of methylation among stromal progenitor cells from ascites, cancerous tissues, and bulk tumor cells. [file 12967_2015_722_MOESM5_ESM.docx]

Table S4. Frequency of methylation among stromal progenitor cells from ascites, cancerous tissues, and bulk tumor cells

| Code No | OCSPCs from ascites | OCSPCs from tissues | Bulk tumors | ρ value  (OCSPCs from tissues vs bulk tumors) |
| --- | --- | --- | --- | --- |
| DLC1 | 7/16(44%) | 4/16(25%) | 3/8(38%) | 0.525 |
| RASS382 | 7/16(44%) | 2/16(13%) | 4/8(50%) | 0.046 |
| CDH13 | 0/16(0%) | 0/16(0%) | 2/8(25%) | 0.037 |
| BRCA1 | 2/8(25%) | 0/16(0%) | 2/8(25%) | 0.037 |
| TIMP3 | 4/16(25%) | 2/16(13%) | 0/8(0%) | 0.396 |
| HIN-1 | 4/16(25%) | 2/16(13%) | 2/8(25%) | 0.439 |
| ESR1 | 2/16(13%) | 2/16(13%) | 0/8(0%) | 0.296 |
| CDKN2A | 2/16(13%) | 1/16(6%) | 2/8(25%) | 0.19 |
| CCND2142 | 5/16(31%) | 8/16(50%) | 3/8(38%) | 0.562 |
| CDKN2B | 8/16(50%) | 4/16(25%) | 5/8(63%) | 0.073 |
